# Editorial: How Reproductive History Influences Our Breast Cancer Risk

**DOI:** 10.3389/fonc.2017.00289

**Published:** 2017-12-04

**Authors:** Robin L. Anderson, Wendy V. Ingman, Kara L. Britt

**Affiliations:** ^1^Metastasis Research Laboratory, Olivia Newton-John Cancer Research Institute, Heidelberg, VIC, Australia; ^2^School of Cancer Medicine, LaTrobe University, Bundoora, VIC, Australia; ^3^School of Medicine at The Queen Elizabeth Hospital, University of Adelaide, Adelaide, SA, Australia; ^4^Robinson Research Institute, University of Adelaide, Adelaide, SA, Australia; ^5^Breast Cancer Risk and Prevention, Peter MacCallum Cancer Centre, Melbourne, VIC, Australia; ^6^The Sir Peter MacCallum Department of Oncology, University of Melbourne, Parkville, VIC, Australia

**Keywords:** estrogen, breast cancer risk, pregnancy, parity, lymphangiogenesis, obesity

**Editorial on the Research Topic**

**How Reproductive History Influences Our Breast Cancer Risk**

Reproductive history has profound effects on women’s breast cancer (BCa) risk. With fertility rates falling, the age of childbearing increasing, and the age of menarche decreasing each decade, it is critically important that we define the biological pathways linking reproductive history to BCa risk. In this special series on reproductive history and breast cancer risk, we hear from six groups who have expertise in this area and share their thoughts on the most pressing questions in the field.

Dall and Britt have an interest in the effects of hormones and reproductive events (menarche, parity, menopause) on breast cancer risk. They describe the increased risk of breast cancer in nulliparous nuns and discuss the current studies attempting to define the role of the mammary stem cells, ER+ cells, and growth factors in parity-induced protection (1, 2). In contrast to the decreased risk that comes with pregnancy, a woman’s risk of ER+ BCa increases is she is older when she enters menopause (3, 4). Polymorphisms in the ER gene, ER signaling (5, 6), DNA damage/repair, and FSH and immune components (7–10) have been associated with the age at menopause but together still only explain a small portion of the timing. As mice do not undergo a natural menopause, experimental data on menopause and breast cancer are lacking. The other reproductive time point to influence cancer risk is the age at menarche; a younger age at menarche increasing BCa risk. This is worrying as the age of menarche has declined from 16.5 years in the 1800s, 13.5 in the early 1900s, to 12 years today. Biologically, menarche begins in response to rising circulating hormones including estrogen, and elevated estrogens have been found in girls experiencing precocious menarche (11). Despite this, genome-wide sequencing studies failed to find a strong association with estrogen-regulated genes (9, 12, 13). Hormone replacement therapy and oral contraceptive pill use both increased BCa risk in current users and can have more detrimental effects on younger users (14, 15). Together, this work highlights that the young breast is particularly sensitive to hormonal changes.

Atashgaran and colleagues study the influence of the reproductive cycle on immune cells within the breast and how this might relate to the increased risk of BCa in women who cycle for extended periods. They explain the effects of fluctuating estrogen and progesterone (during menstrual cycling) on the mammary epithelial cells, immune cells, and extracellular matrix. The ability of transformed cells to evade the immune response is a hallmark of cancer (16). Fluctuations in hormones during the mouse estrous cycle alters the abundance, phenotype, and function of local macrophages (17, 18), which can affect their ability to recognize DNA-damaged cells, phagocytose them, and generate adaptive immune responses (18). In particular, progesterone regulates the Th1/Th2 phenotypes of T cells in the mammary gland (19) and induces Th2 cytokines during pregnancy (20). Th1 cytokines are thought to mediate antitumor immunity and tumor rejection, whereas Th2 cytokines are produced by tumors and are involved in pro-tumorigenic responses (21, 22). Estradiol induces a pro-inflammatory cytokine profile in the estrus phase that can be mitigated by progesterone during other phases of the cycle (23). It is possible that aberrant hormonal exposure can negatively influence the inflammatory milieu of the breast and aid in tumor development and cancer progression.

Katz and her team have been trying to understand the role of insulin-like growth factors (IGF) in parity-induced protection against breast cancer. They dissected mammary glands from parous mice and age-matched virgins and a found Igf1r to be hypermethylated and silenced in parous mammary glands (2) aligning with the fact that high IGF1 levels are associated with increased BCa risk (24, 25). Parous mice, with a low tumor incidence (16%) compared to nulliparous mice (100%) treated with carcinogen, had their protective effect eliminated if they were first treated with IGF (83%) (26). This work is supported by a recent DNA methylation study performed on parous and nulliparous women showing that the IGF acid labile subunit (responsible for transport of IGF1 in the circulation) was hypomethylated with parity (27). Unfortunately, targeting the IGF pathway using IGF1R inhibitors leads to toxic effects that will limit their use as a preventative (28–30); however, this work highlights the importance of exploring other options to promote the parity-associated breast cancer protection.

While there is a lot of interest in trying to define the role of hormones in parity-induced protection, prior to the protection instilled by parity, each woman passes through a transient increased risk period. Borges and colleagues have been studying this postpartum period of mammary gland involution, characterized by regression and remodeling of the epithelium. Postpartum BCa is diagnosed within 5 years of a woman’s most recent childbirth (31, 32), and these cancers tend to be of poor prognosis with an increased likelihood to metastasize (31–34). Similarly, tumor cells implanted into postpartum murine hosts have increased growth, invasive and metastatic capacities compared to those implanted in nulliparous controls (35–39). This environment is driven by immunosuppression and lymphangiogenesis and, here, Borges and colleagues review the role for lymphangiogenesis, the outgrowth of new lymphatic vessels (40–42). New lymphatic formation or neo-lymphangiogenesis occurs in adult tissues in response to infection, inflammation, and wound healing and is stimulated by vascular endothelial growth factors within the damaged tissue. The growth factors VEGF-A, -C, and -D produced by the local fibroblasts, inflammatory cells, and macrophages bind to their receptors on nearby lymphatic endothelial cells and cause the lymphatics to expand (43–47). Borges et al. have shown that neo-lymphangiogenesis occurs during postpartum mammary gland along as does an upregulation of VEGF-C, VEGF-D, and their receptors (36). This increase in lymphatic vessel density in the postpartum breast (1–5 years post pregnancy) may explain the increased likelihood of tumor development in these women who make up a large proportion of all BCa diagnosed in young (<45 years) Caucasian women (33).

Au and colleagues work on the role of adipose-derived estrogen in breast cancer development. Local estrogen production (through the enzyme aromatase) is increased in tumor-bearing breast tissue and systemic antiestrogen therapies are widely used to target this in ER+ BCa patients. These hormonally driven BCa are more prevalent in postmenopausal women where an increased BMI has been associated with increased local breast estrogen (48–50). In these women, the adipose tissue (rather than the ovaries) is the primary site of estrogen production (51). Aromatase is increased in breast adipose tissue of obese women as a number of obesity-associated factors (inflammatory mediator PGE2 and the adipokine leptin) can stimulate aromatase expression (52, 53). Au and colleagues have tried to find a factor that inhibits aromatase expression in the adipose as a novel targeted antiestrogen therapy. They found that the gut-derived hormone ghrelin (produced in the stomach to regulate appetite and growth hormone release) inhibits aromatase expression in adipose cells (54, 55). In addition, circulating levels of ghrelin, and its unacetylated form des-acyl ghrelin, are lower in obese women and cannot function as well to inhibit estrogen-driven BCa growth. They describe the work they are now doing on Ghrelin/des-acyl ghrelin mimetics as alternative endocrine therapeutics.

In order to define how aggressive a tumor is and possibly identify if it would be responsive to endocrine therapy, clinics are adopting gene-expression profiling on the tumors to provide an intrinsic, molecular portrait of the tumor and identify the likelihood of recurrence. Bernhardt and colleagues are concerned that these tests have been developed using tumor tissue from postmenopausal women (non-cycling) but are being used for women at all ages. Of the gene expression tests available (PAM50, Oncotype DX and EndoPredict) were developed in postmenopausal women and now not performing well in premenopausal women who make up 25% of all patients (56–58). It is postulated that this is because the tests are largely reliant on proliferation and estrogen signaling genes, which are expected to differ markedly in pre- and postmenopausal women. The former undergo cyclical production of ovarian hormones which drive proliferation, differentiation, and apoptosis through gene expression changes (59, 60). These hormonally driven changes are not present in the postmenopausal women. Supporting these concerns, it has been found that the MammaPrint gene expression test that was developed and validated in women under 55 years of age (61, 62) is performing well for younger women. This suggests that for the most success in guiding clinical treatment, gene-expression tests should be developed in the women for which we intend to use them.

## Author Contributions

RA: this author assisted in the design and was involved in reviewing articles. Assisted with drafting the editorial and approved final version. WI: this author wrote two review articles and was involved in reviewing articles. She assisted with drafting the editorial and approved final version. KB: this author was the lead editor and conceived the special issue. Invited all contributors, wrote a review article, and assisted with reviewing articles. Wrote the editorial.

## Conflict of Interest Statement

The authors declare that the research was conducted in the absence of any commercial or financial relationships that could be construed as a potential conflict of interest.
